# AmpC and extended spectrum beta-lactamases production among urinary isolates from a tertiary care hospital in Lalitpur, Nepal

**DOI:** 10.1186/s13104-017-2784-5

**Published:** 2017-09-07

**Authors:** Suman Rai, Narayan Dutt Pant, Raju Bhandari, Anil Giri, Roshan Parajuli, Manoj Aryal, Jyoti Amatya, Vijay Kumar Sharma

**Affiliations:** 1 Department of Microbiology, Tri-Chandra Multiple Campus, Kathmandu, Nepal; 2 Department of Microbiology, Grande International Hospital, Kathmandu, Nepal; 3 Department of Microbiology, Goldengate International College, Kathmandu, Nepal; 4 Department of Microbiology, St. Xaviers College, Kathmandu, Nepal; 5 Institute of Medicine, Tribhuvan University, Kathmandu, Nepal

**Keywords:** Urinary tract infection, ABL, ESBL, Co-production, Nepal

## Abstract

**Background:**

Production of AmpC and extended spectrum beta-lactamases among urinary isolates has created a serious problem to the successful management of the urinary tract infection. The main purpose of this study was to determine the rates of the extended spectrum beta-lactamase (ESBL) production and AmpC beta-lactamase (ABL) production among urinary isolates.

**Results:**

Among total 564 urinary isolates, 514 (91.1%) were gram negative bacilli and 50 (8.9%) were gram positive cocci. *E. coli* (76.1%) was the most common bacteria isolated. *Staphylococcus aureus* (6.7%) was the predominant gram positive bacteria isolated. 35 (6.8%) of the 514 gram negative bacilli were ESBL producers. Similarly, 14 (2.7%) of the gram negative bacilli were ABL producers. Only one isolate was ESBL and ABL co-producer. Highest rate of susceptibility of gram negative bacteria was seen toward amikacin (97.3%) followed by imipenem (94.4%). Similarly, highest rate of susceptibility among gram positive cocci was seen toward vancomycin (100%) followed by amikacin (93.5%).

**Conclusions:**

Low rates of AmpC and extended spectrum beta-lactamases production in comparison to other previous studies were reported. On the basis of the antimicrobial susceptibility patterns of the bacteria we reported in our study, amikacin, imipenem and nitrofurantoin can be used for the preliminary treatment of urinary tract infections caused by gram negative bacteria and vancomycin and amikacin for treatment of urinary tract infections caused by gram positive bacteria.

## Background

Urinary tract infection is among the commonly encountered bacterial infections in human [1]. Antibiotics are important therapeutic means for the treatment of bacterial infections. However, the development of drug resistance among the bacteria has created a serious problem to the successful treatment of the bacterial infections. Production of AmpC and extended spectrum beta-lactamases are the important mechanisms of drug resistance mainly among gram negative bacteria. All ESBL producing organisms should be considered resistant to all penicillins (except temocillin), cephalosporins (except cefoxitin and cefotetan) and aztreonam [2, 3]. So, there are limited treatment options for infections caused by extended spectrum beta-lactamase (ESBL) producing organisms and their involvement in severe infections may result into treatment failure if the antibiotics are chosen just on the basis of regular antimicrobial susceptibility testing without determination of ESBL production [3]. ESBL producing bacteria may show in vitro susceptibility to some extended spectrum cephalosporins but the use of such antibiotics for treatment of the infections by ESBL producing strains may result into treatment failure [3]. Similarly, AmpC β-lactamases producing organisms are resistant to narrow, broad-, and expanded-spectrum cephalosporins and cephamycins and can not be inhibited by clavulanate, sulbactam, and tazobactam [4]. They show non-beta-lactam coresistance and have a few treatment options [5]. Further, they have high potential to transfer the drug resistance features to other bacteria horizontally [5]. In Nepal, limited information is present in AmpC beta-lactamase (ABL) producing bacteria in comparison to extended-spectrum β-lactamase-producing organisms [5]. Different studies in Nepal have reported the different rates of ESBL and ABL production among gram negative bacteria [1, 5]. So in this study we determined the prevalence of the ESBL and ABL producing organisms in causing urinary tract infection.

## Methods

### Study design and setting

A descriptive cross-sectional hospital based study was conducted using all 564 urinary isolates isolated from April 2014 to October 2014 at Microbiology Laboratory of Alka Hospital, Lalitpur, Nepal. The isolates were already collected and stored.

### Identification of the organisms

The organisms were identified using colony morphology, Gram’s stain and biochemical tests [6, 7]. The common biochemical tests used were oxidase test, catalase test, sulphur indole motility test, urease test, citrate utilization test, tripal sugar iron agar test, methyl red and Voger-Proskauer test, coagulase test, bile esculin agar test etc.

### Antimicrobial susceptibility testing

The antimicrobial susceptibility testing was performed using Kirby-Bauer disc diffusion technique following clinical and laboratory standards institute guidelines [8].

### Detection of ESBL and ABL production

Detection of ESBL production was done by combined disc method using ceftazidime and ceftazidime/clavulanic acid discs [8] and detection of ABL production was done by using cefoxitin and cefoxitin/boronic acid discs [9].

## Results

Among total 564 bacteria, 514 (91.1%) were gram negative bacilli and 50 (8.9%) were gram positive cocci. *E. coli* (76.1%) was the most common bacteria followed by *Staphylococcus aureus* (6.7%), *Klebsiella pneumoniae* (4.6%), *Proteus mirabilis* (2.3%), *Citrobacter freundii* (2.3%), *Providencia* spp. (1.6%), *Enterococcus faecalis* (1.6%), *Morganella morganii* (1.4%), *Acinetobacter* spp. (1.1%), *Proteus vulgaris* (0.9%), coagulase negative *Staphylococcus* spp. (0.6%), *Klebsiella oxytoca* (0.4%) and *Pseudomonas aeruginosa* (0.2%).

### Production of ESBL and ABL by gram negative urinary isolates

35 (6.8%) of the 514 gram negative bacilli were ESBL producers. Among ESBL producing gram negative bacilli, 33 were *E. coli* and 2 were *K. pneumoniae*. Similarly, 14 (2.7%) of the gram negative bacilli were ABL producers. Among which, 10 were *E. coli* and 1 each *K. pneumoniae, C. freundii, K. oxytoca* and *M. morganii.* Only one isolate was ESBL and ABL co-producer.

### Antibiotic susceptibility patterns of gram negative bacilli

Highest rate of susceptibility was seen toward amikacin (97.3%) followed by imipenem (94.4%) (Table 1).

**Table 1:** Antibiotic susceptibility patterns of gram negative bacilli (n = 514)

Antibiotics	Susceptibility (%)
Amoxicillin (20 µg)	11.2
Ciprofloxacin (5 µg)	62.7
Norfloxacin (10 µg)	61.5
Ofloxacin (5 µg)	63.3
Nitrofurantoin (300 µg)	85.6
Nalidixic acid (30 µg)	34.9
Cotrimoxazole (25 µg)	50.9
Cefixime (5 µg)	62.2
Ceftriaxone (30 µg)	68.1
Amikacin (30 µg)	97.3
Imipenem (10 µg)	94.4
Piperacillin/tazobactam (110 µg)	72.9
Cefoperazone/sulbactam (85 µg)	71.8

### Antibiotic susceptibility patterns of gram positive cocci

Highest rate of susceptibility was seen toward vancomycin (100%) followed by amikacin (93.5%) (Table 2).

**Table 2:** Antibiotic susceptibility patterns of gram positive cocci (n = 50)

Antibiotics	Susceptibility (%)
Amoxicillin (20 µg)	41.9
Ciprofloxacin (5 µg)	63.3
Ofloxacin (5 µg)	68.2
Cotrimoxazole (25 µg)	46.8
Erythromycin (15 µg)	50
Cloxacillin (1 µg)	38.5
Vancomycin (5 µg)	100
Amikacin (30 µg)	93.5
Cefixime (5 µg)	36.8
Ceftriaxone (30 µg)	70

## Discussion

As in our study, Bhatt et al. [10] also found the gram negative bacilli to be higher in number among urinary isolates (92.3%) followed by gram positive cocci (7.7%) with *E. coli* being most common organism. In our study, 6.8% and 2.7% of all gram negative bacilli isolated were found to be ESBL and ABL producers, while one isolate was ESBL and ABL co-producer. However, Chander and Shrestha reported higher rate of ESBL production among uropathogens in Nepal [11]. Similarly, Ansari et al. [12] found the rates of ABL production (9%) and ESBL/ABL co-production (4%) to be higher. The prevalence of drug resistance among clinical isolates due to different mechanisms varies from place to place and is changing with time. The main reason for this may be difference in the antibiotic usage in different area during different period of time. The co-expressions of different variants of ESBL and AmpC genes may be responsible for AmpC/ESBL co-production [13] but sometime this co-production may give false negative tests in the detection of ESBL [13]. In this study, highest rate of susceptibility among gram negative isolate was seen toward amikacin followed by imipenem and nitrofurantoin. Similarly, Noor et al. reported highest rate of susceptibility toward imipenem followed by amikacin among uropathogens [14]. Neupane et al. also found the highest rate of susceptibility toward amikacin (87.5%) followed by nitrofurantoin (72.6%) [15]. High rate of susceptibility to nitrofurantoin was also noted by Bhatt et al. (82.2%) [10]. Though the bacteria show high susceptibility to these drugs, amikacin belonging to aminoglycosides which have nephrotoxic effect and imipenem being an intravenous antibiotic, they are not recommended as the first line therapy in the treatment of urinary tract infection. Further, nitrofurantoin is recommended only for the treatment of uncomplicated urinary tract infection because it does not give good tissue concentration [16].

Among gram positive cocci, highest rate of susceptibility was seen toward vancomycin (100%) followed by amikacin (93.5%). No vancomycin resistant *S. aureus* isolates have been reported from Nepal [17].

## Limitations of the study

Due to lack of resources and limited time available we could not use molecular techniques and large numbers of samples in our study.

## Conclusion

Low rates of AmpC and extended spectrum beta-lactamases production in comparison to other previous studies were reported. On the basis of the antimicrobial susceptibility patterns of the bacteria we reported in our study, amikacin, imipenem and nitrofurantoin can be used for the preliminary treatment of urinary tract infections caused by gram negative bacteria and vancomycin and amikacin for the treatment of urinary tract infections caused by gram positive bacteria.

## Abbreviations

ABL: AmpC beta-lactamase
ESBL: extended spectrum beta-lactamase
